# Monitoring Quality Across Home Visiting Models: A Field Test of Michigan’s Home Visiting Quality Assurance System

**DOI:** 10.1007/s10995-018-2538-6

**Published:** 2018-06-05

**Authors:** Julia Heany, Jennifer Torres, Cynthia Zagar, Tiffany Kostelec

**Affiliations:** 1 Michigan Public Health Institute, Center for Healthy Communities, 2342 Woodlake Drive, Okemos, MI 48864 USA; 2 Michigan Public Health Institute, Public Health Services, 2364 Woodlake Drive, Suite 180, Okemos, MI 48864 USA; 3 Michigan Department of Health and Human Services, 109 W. Michigan Ave, 7th FL, Lansing, MI 48933 USA

**Keywords:** Home visiting, Implementation quality, Quality assurance, Quality improvement

## Abstract

*Introduction* In order to achieve the positive outcomes with parents and children demonstrated by many home visiting models, home visiting services must be well implemented. The Michigan Home Visiting Initiative developed a tool and procedure for monitoring implementation quality across models referred to as Michigan’s Home Visiting Quality Assurance System (MHVQAS). This study field tested the MHVQAS. This article focuses on one of the study’s evaluation questions: Can the MHVQAS be applied across models? *Methods* Eight local implementing agencies (LIAs) from four home visiting models (Healthy Families America, Early Head Start-Home Based, Parents as Teachers, Maternal Infant Health Program) and five reviewers participated in the study by completing site visits, tracking their time and costs, and completing surveys about the process. LIAs also submitted their most recent review by their model developer. The researchers conducted participant observation of the review process. *Results* Ratings on the MHVQAS were not significantly different between models. There were some differences in interrater reliability and perceived reliability between models. There were no significant differences between models in perceived validity, satisfaction with the review process, or cost to participate. Observational data suggested that cross-model applicability could be improved by assisting sites in relating the requirements of the tool to the specifics of their model. *Discussion* The MHVQAS shows promise as a tool and process to monitor implementation quality of home visiting services across models. The results of the study will be used to make improvements before the MHVQAS is used in practice.

## Significance

Given the importance of implementation quality and fidelity, states responsible for administering grant funding to home visiting programs have the potential to play a critical role in ensuring that funds are directed toward agencies that implement quality programs and produce positive outcomes. However, few tools exist to assure and improve quality implementation of home visiting models across a system. This field test of Michigan’s Home Visiting Quality Assurance System contributes to the limited evidence base on measuring the quality of home visiting program implementation by testing a tool that assesses quality and fidelity across models and informs decision making.

## Introduction

The positive impact of evidence-based home visiting models on family wellbeing has become more broadly understood since federal Maternal Infant Early Childhood Home Visiting (MIECHV) funding was authorized in 2010. MIECHV and other efforts to take evidence-based models to scale are driven by the assumption that evidence-based models are more likely to achieve intended outcomes than models that are untested. However, as evidence-based models transition from the well-controlled context of a research study to a more dynamic real world context, fidelity to the original model can be challenging.

The variability observed in the outcomes of home visiting evaluation studies has been attributed, in part, to lack of model adherence (Howard and Brooks-Gunn 2009). Effective implementation and maintaining model fidelity drive better outcomes (Carroll et al. 2007; Donelan-McCall et al. 2009; Russell et al. 2007), and attention to implementation is critical to ensuring effective and efficient use of programming dollars (Fixsen et al. 2005).

Each home visiting model establishes, reviews, and updates its own criteria for fidelity based on the experience and expertise of developers and implementers. In contrast, efforts to identify criteria critical for achieving outcomes across different models are limited. However, as part of the Supporting Evidence-Based Home Visiting to Prevent Child Maltreatment (EBHV) initiative, Daro (2010) and Mathematica Policy Research developed a concise set of quantitative measures of home visiting fidelity that they applied across five models and 46 implementing agencies. The study found significant variability in model fidelity, and the authors concluded that it was both possible and important for state administrators of home visiting funds to monitor implementation across models. Additionally, Korfmacher et al. (2012) developed and are testing a Home Visiting Program Quality Rating Tool (now titled Prevention Initiative Quality Rating Instrument), designed to work across home visiting models that is grounded in the literature regarding what works in home visiting. Although reliability and validity testing were still in process and the authors cautioned against using the Tool until more testing and development was completed, initial testing suggested it was possible to establish useful quality indicators across home visiting models to inform improvement efforts.

Both studies suggested that states responsible for administering grant funding to home visiting programs are in a position to play a critical role in monitoring implementation and assuring program quality. States can establish expectations for quality and fidelity, and they can provide direct support for quality improvement. Through the Home Visiting Collaborative Improvement and Innovation network (HV CoIIN) and state-driven efforts, home visiting as a field is building its capacity in using quality improvement methods across models. However, there have been relatively few federal or state-level efforts to establish cross-model quality standards and quality assurance processes, and there has been little research focusing on building practical systems for monitoring and improving quality in home visiting model implementation. Systems for assessing the quality of similar services, such as those provided by community health workers, have been developed and tested nationally and internationally, and found to support quality service delivery (Crigler et al. 2013). However, these systems are not directly applicable to cross-model quality assurance in a home visiting context.

Building on early work to develop cross-model quality indicators and monitoring processes, the current study tested a comprehensive quality assurance tool designed to assess quality and fidelity across home visiting models and support quality improvement. Michigan’s Home Visiting Quality Assurance System (MHVQAS) tool was developed as part of Michigan’s MIECHV initiative. In Michigan, MIECHV funds 17 Local Implementing Agencies (LIAs) who served 1963 families in fiscal year 2016. More broadly, state funded home visiting programs served over 34,000 families in that same year. Following validation of the tool, Michigan plans to require that all home visiting programs funded with MIECHV funds, or those who receive specifically-identified home visiting funding from the Michigan Department of Health and Human Services, are reviewed with the MHVQAS at least once every 3 years. Home visiting models will include Healthy Families America, Early Head Start, Parents as Teachers, and Nurse Family Partnership.

The study contributes to the limited but growing evidence base on measuring the quality of home visiting program model implementation by testing the validity and reliability of the tool, and examining the ability of the tool to assess quality and fidelity across models and inform decision making. In addition to this contribution to the literature, the MHVQAS has implications for policy, in that the tool provides a framework for a system-wide definition of quality and a mechanism for monitoring quality across home visiting models. A system-wide definition and tool could help states identify statewide support and technical assistance policies and practices that would aid the entire system, and identify opportunities for state-level quality improvement projects. The study aimed to: (1) determine if the MHVQAS tool produces valid and reliable results across models, and (2) determine if the MHVQAS procedure can be feasibly implemented and used to support quality improvement. This article focuses on one of the evaluation questions from the study: Can the MHVQAS be applied across models implemented in Michigan?

## Methods

### Participants

This mixed methods study conducted a field test of the MHVQAS tool and process, examining validity, reliability, costs, and utility. Five reviewers were enrolled into the study and trained to conduct reviews of local home visiting programs using the MHVQAS tool and process. Each reviewer had expertise in a different home visiting model: Healthy Families America (HFA), Parents as Teachers (PAT), Early Head Start (EHS), Nurse Family Partnership (NFP), and Maternal Infant Health Program (MIHP). Eight home visiting LIAs, with a total of 91 staff, were enrolled into the study to participate in the MHVQAS review process (two each of: HFA, PAT, EHS, and MIHP). The researchers recruited NFP LIAs, but were unable to enroll any LIAs from that model. LIAs were chosen through model national office recommendations, based on the following inclusion criteria: success in implementing their model’s fidelity indicators, a review by their model developer within the past two years or within the coming year, and use of a data system that captured basic information about implementation.

### Data Collection and Analysis

#### MHVQAS Tool and Site Visits

The MHVQAS tool was developed through review of model requirements from evidence-based home visiting models, the research literature, MIECHV benchmarks and constructs, and existing instruments for monitoring quality (Daro 2010; Korfmacher et al. 2012), along with discussion with experts in the field. The tool was organized into eight domains, 19 standards, and 72 measures. Domains are broad categories that relate to an element of implementation quality (e.g., home visit content). Standards are expectations for quality under each domain (e.g., use of evidence-informed content). Measures define how performance will be assessed under each standard (e.g., policy that describes use of evidence-informed content). Measures within each standard require home visiting LIAs to demonstrate that they have put in place written policy or procedures (for standards 1–17) and can demonstrate practice that aligns with model requirements, if applicable. Each measure is rated as fully met (i.e., meet the expectation), partially met (i.e., opportunity for improvement), or unmet (i.e., improvement plan needed). The criteria for ratings are specific to each measure. A list of domains and standards can be found in Table 1. An example of a measure can be found in Table 2.

**Table 1:** MHVQAS domains and standards

Recruitment and enrollment
Standard 1: Home visiting implementing sites recruit and enroll families that meet eligibility criteria
Home visitor and supervisor caseloads
Standard 2: home visiting implementing sites maintain appropriate home visitor caseloads
Standard 3: home visiting implementing sites maintain appropriate supervisor caseloads
Assessment of family needs and referral to services
Standard 4: home visiting implementing sites assess family needs and provide referrals when appropriate
Standard 5: home visiting implementing sites conduct developmental screenings and provide referrals when appropriate
Dosage and duration
Standard 6: home visiting implementing sites provide home visits with the frequency and duration necessary to achieve intended outcomes for families
Standard 7: home visiting implementing sites retain families until they complete services and support families as they exit the program
Home visit content
Standard 8: home visiting implementing sites individualize program delivery to family risks and needs, as well as family strengths and protective factors
Standard 9: home visiting implementing sites use evidence-informed content/curriculum/curricula
Standard 10: home visiting implementing sites build positive and productive relationships between home visitors and families
Staff qualifications and supervision
Standard 11: home visiting implementing sites are staffed by qualified supervisors
Standard 12: home visiting implementing sites are staffed by qualified home visitors
Standard 13: home visiting implementing sites provide home visitors with supervision that reduces the emotional stress of home visiting, reduces burnout and turnover, and improves performance
Standard 14: home visiting implementing sites provide supervisors with supervision that improves their skill and effectiveness
Professional development
Standard 15: home visiting implementing sites provide staff with the training necessary to deliver the program as designed
Organizational structure and support
Standard 16: home visiting implementing sites receive guidance and support from partners
Standard 17: home visiting implementing sites have the infrastructure necessary to support high quality implementation
Standard 18: home visiting implementing sites assure and improve program quality
Standard 19: home visiting sites are integrated within the broader service system for children and families in their communities

**Table 2:** Example of MHVQAS measure

Standard 9: home visiting Implementing Sites use evidence-informed content/curriculum/curricula			
Measure	Expectation and required components	Review procedure	Rating scale
The home visiting program has a policy that describes the use of evidence-informed content/curriculum/curricula used by home visitors and how it will be incorporated into visit plans. Policy shall reflect model expectations, if applicable	The site will provide documentation that describes The content that is delivered during home visits—must align with model requirements, if applicable: see model guidance Procedures for incorporating content into visit plans, including procedures for modifying the order, eliminating components, and adding components Expectations for covering the content over the course of service	The site will provide documentation that describes the evidence-informed content covered during home visits. The site reviewer will assess the written documentation for each required component. The site reviewer will assess the degree to which the policy aligns with model expectations. If necessary the site reviewer will ask the site for clarification during the site visit	3—Fully met, all three components are clear, complete, and aligned with model expectations 2—Partially met, fewer than three components are clear, complete, and aligned with model expectations 1—Unmet, policy does not exist, does not meet model expectations, or does not describe the use of evidence-informed content that is delivered during home visits

The tool was designed to be completed by trained reviewers. For the field study, trained reviewers completed review of documentation and data prior to and during a daylong site visit. During the site visit, reviewers examined client files, supervision documentation, and meeting minutes. They also received a tour of the facility and interviewed program administrators, supervisors, and home visitors. Reliability of review procedures was supported through training and use of standardized materials such as interview questions and worksheets.

Two reviewers scored the LIA independently and did not discuss ratings. After each site visit, both reviewers independently completed a Quality Report that summarized their ratings. Reviewers then met to compare their ratings, and come to consensus by discussing notes they had made, referencing back to the MHVQAS tool, and seeking understanding in how their counterpart made their decision. Consensus was consistently met through these means. After consensus, the final Quality Report was sent to the LIA, with suggestions for improvement on measures that were ‘partially met’ or ‘unmet,’ and the reviewers discussed the Quality Report with the LIA by phone conference. All LIAs received a copy of the MHVQAS before the site review, with detailed descriptions of the requirements for each measure and the criteria used to determine the ratings.

MHVQAS tool ratings were compared across models by computing a mean score for each LIA for each standard. Given the small sample size of four models, and the fact that ratings were not normally distributed, scores were compared across models using a Kruskal–Wallis H Test. Interrater reliability was analyzed by computing the Cohen’s Kappa between the two reviewers’ ratings on each measure (n = 72) for each site visit. To examine differences in interrater reliability between models, the Cohen’s Kappa table was combined for the two LIAs in a model, and compared with the combined Cohen’s Kappa tables of the LIAs from the other models.

### Observation

The researchers observed key parts of the review process, including site visits, consensus meetings between reviewers, and calls with LIAs to review the Quality Report. During these observations, fieldnotes were taken on aspects of the process that went well, and aspects that created challenges. Fieldnotes were analyzed by two members of the research team, who independently reviewed fieldnotes and identified common themes related to reliability, validity, usefulness of the MHVQAS process, and cross-model applicability. They then met to compare themes, and worked together to come to consensus.

### Surveys

A 48-item Reviewer Satisfaction Survey was sent after each of the eight site visits to the two reviewers for each LIA (n = 16, response rate 100%). It assessed factors such as the ease of making decisions about whether indicators were met, strengths and limitations of the tool, and the efficiency of the process.

A 50-item LIA Staff Satisfaction Survey was sent to home visiting staff after each site visit (n = 69, response rate 83.1%). The survey assessed factors such as the efficiency of the process, strengths and limitations of the tool, and the likelihood that the LIA would use findings to inform improvement.

Face validity of the tool was analyzed using scales of three items from the LIA Staff Satisfaction Survey (Cronbach’s α = 0.833) and two items from the Reviewer Satisfaction Survey (α = 0.836) that asked about perceptions of the tool related to validity (e.g., “The standards reflect key drivers of quality in home visiting”) using a Likert scale ranging from 1 (strongly disagree) to 6 (strongly agree). Perceived reliability was analyzed using scales of 13 items from the LIA Staff Satisfaction Survey (α = 0.978) and 13 items from the Reviewer Satisfaction Survey (α = 0.979) that asked about perceptions of the tool and guidance document related to reliability (e.g., “The measures to be assessed are clear”) using a Likert scale ranging from 1 (strongly disagree) to 6 (strongly agree). Mean scale scores for face validity and perceived reliability were compared between the four models using analysis of variance.

Each LIA was invited to complete a LIA Staff 6 Month Follow-Up Survey (n = 22, response rate 100% of LIAs) that focused on how results were communicated and to whom, which findings were helpful and unhelpful, and if/how findings were used to inform improvement activities. Usefulness of the process was examined using a scale of three items (α = 0.739) that asked about whether the Quality Report had informed decision making (e.g., “The ‘opportunities for improvement’ described in the report were actionable”) using a Likert scale ranging from 1 (strongly disagree) to 6 (strongly agree). Mean scale scores for usefulness of the process were compared between the four models using analysis of variance.

Open ended questions from the surveys were analyzed using content analysis. Two members of the research team independently reviewed answers and identified explicit and implicit content related to reliability, validity, usefulness, and cross-model applicability. They then used an inductive analysis to identify key themes for each content area. The two researchers then met to compare themes, and worked together to come to consensus by discussing similarities and differences between themes, and returning to the data to refine themes as needed.

One important aspect of the feasibility of implementing a quality assurance process is cost. Therefore, a Cost Tracking Survey was sent after each of the eight site visits to the two reviewers for each LIA (n = 13, response rate 81.3%) and to all home visiting staff (n = 68, response rate 81.9%). Cost Tracking Surveys were used to calculate the total cost for each site, including staff time and additional costs, and the mean number of hours spent for reviewers. Mean total time and mean total cost of time were compared between the four models using analysis of variance. All surveys were administered through Qualtrics, an online survey software.

### Model Review Reports

**Fig. 1:**
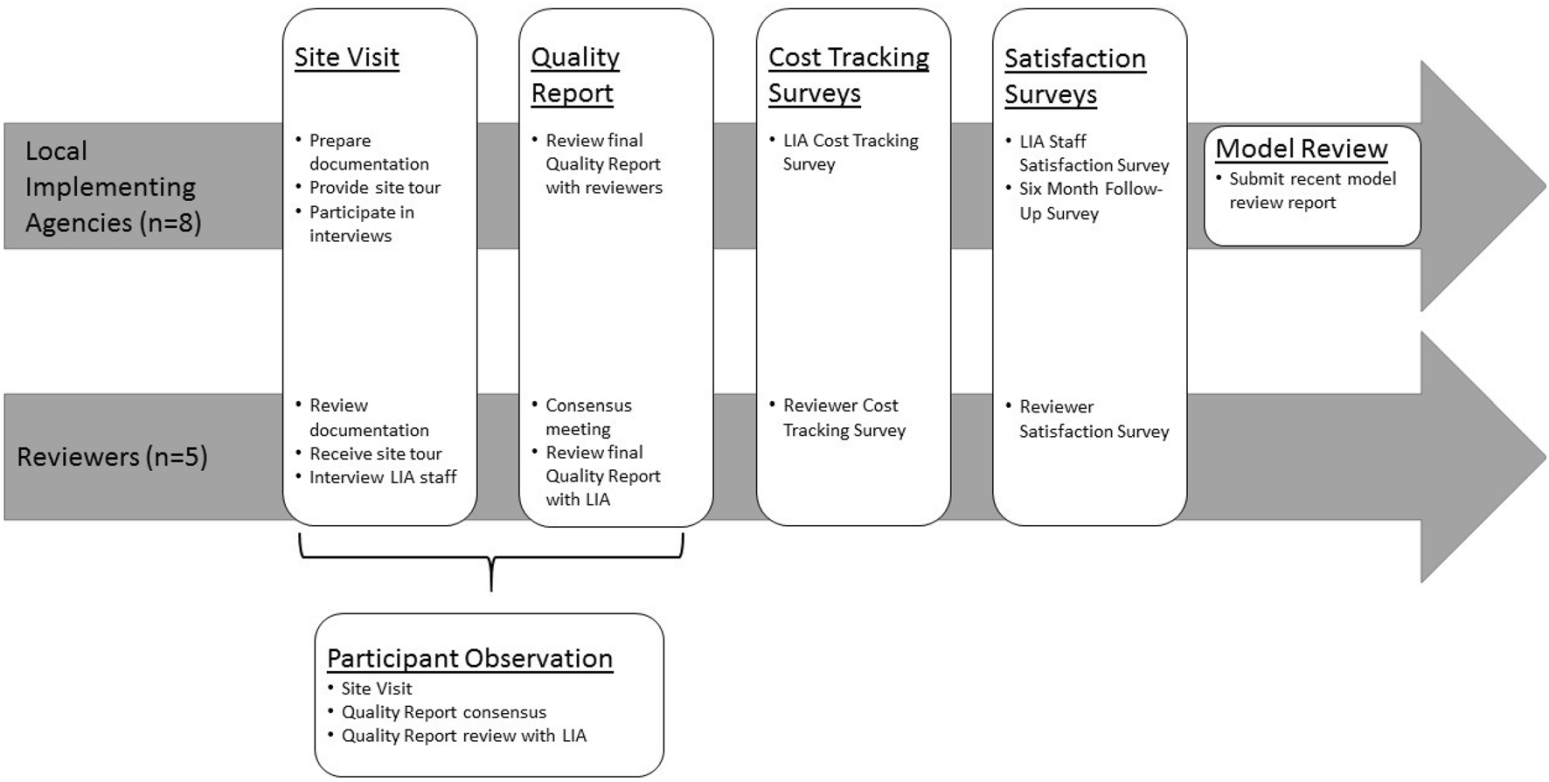
Michigan’s home visiting quality assurance system field study procedures

Each model has a process for reviewing fidelity of implementation, and produces a report that describes their findings. These reports were collected from all eight participating LIAs to assess criterion validity after the site visit and Quality Report were completed. Because reports differ by model, the first step in the analysis of model reviews was to cross-walk the MHVQAS with each model review to identify the underlying concepts measured by each and organize them into categories. Because the MHVQAS is organized into domains, standards, and measures, a similar structure was used to organize model reviews, creating one set of domains and standards that were common across models and could be used for comparison. Once each measure was categorized, overall performance in that area on the model review was compared to overall performance in that area on the MHVQAS review by determining whether both reviews identified strengths or gaps in each category. The researchers then tallied the number of domains where the MHVQAS findings aligned with the model findings for each LIA. Figure 1 shows an overview of the MHVQAS procedures and study procedures.

This study was reviewed and approved by the Michigan Public Health Institute’s Institutional Review Board (IRB) and the Michigan Department of Health and Human Services IRB.

### Results

Summary scores for each MHVQAS standard were compared by model. Kruskal–Wallis H tests showed that there was not a statistically significant difference in ratings on any of the Standards between the different home visiting models (χ^2^(3) ranged from 0.81 to 7.0; *p* ranged from 0.07 to 0.85).

**Fig. 2:**
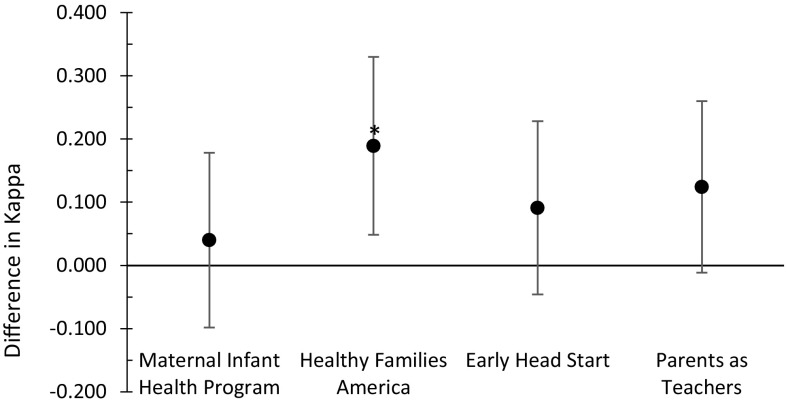
Single model κ scores compared with the other models combined κ. Difference between a single model’s κ and the combined κ of the remaining models. Confidence interval of the difference was used to create error bars. *Confidence interval of the difference does not contain 0

Interrater reliability was compared across models as well. The Cohen’s Kappa (κ) agreement scores for each model ranged from 0.240 (fair agreement) to 0.452 (moderate agreement). The combined κ of the HFA LIAs had higher agreement than the other models (95% CI [0.048, 0.330]). The combined κ of the PAT LIAs had the lowest agreement compared with the other models (see Fig. 2).

Analysis of variance of the LIA Staff Survey Reliability scale showed that there was not a statistically significant difference between the four models (see Table 3). Analysis of variance of the Reviewer Survey Reliability scale showed that there was a statistically significant difference between the four models. A Tukey post-hoc test revealed that reliability for MIHP site reviews (*M* = 3.10, *SD* = 1.033) was significantly lower than HFA (*M* = 5.12, *SD* = 0.263) and PAT (*M* = 5.08, *SD* = 0.548). Analysis of variance of the LIA Staff and Reviewer Validity scales showed that there was a not a statistically significant difference between the four models.

**Table 3:** Differences in reliability, validity, satisfaction, and usefulness of the MHVQAS across home visiting models

	Mean	SD	ANOVA		
			d.f	*F*-value	*p*-value
Reliability of MHVQAS					
LIA Staff (n = 49)	4.49	0.81	3	0.004	1.000
Reviewers (n = 16)	4.44	1.14	3	4.84	0.020
Validity of MHVQAS					
LIA Staff (n = 44)	4.44	0.82	3	0.81	0.496
Reviewers (n = 16)	4.91	0.90	3	1.58	0.247
Satisfaction with MHVQAS					
LIA Staff (n = 35)	4.81	0.62	3	1.68	0.192
Reviewers (n = 16)	4.95	0.46	3	0.36	0.782
Usefulness of MHVQAS					
LIA Staff (n = 21)	4.65	0.60	3	0.88	0.471

Possible scale scores ranged from 1 to 6

Analysis of variance of the LIA Staff and Reviewer Satisfaction scales showed that there was not a statistically significant difference between the four models. Analysis of variance of the 6 Month Follow-Up Survey questions about whether the Quality Report informed decision making showed that there was not a statistically significant difference between the four models.

The cross-walk of the MHVQAS and LIA model reviews identified that, for each of the four models, the MHVQAS included standards that were not included in the model’s own standards. However, each of the MHVQAS standards was included in at least one model. One domain that MIHP and HFA included was not explicitly included in the MHVQAS, which was family rights. While the MHVQAS had a standard around family feedback, MIHP and HFA had additional standards around accommodating families based on specific characteristics, such as language, race/ethnicity, and disability. EHS had additional standards around environmental health and safety, but most of these were specific to center-based services.

Findings from the MHVQAS and model reviews were then compared for those standards that were similar across both systems to assess criterion validity. The MHVQAS review and the model reviews had some differences in ratings in areas where the MHVQAS and models shared similar standards. On average, the MHVQAS findings aligned with the model findings on 87% of the standards. However, there did not appear to be any standards that were systematically rated differently by the MHVQAS.

Observational data and answers to open-ended questions on the Satisfaction Surveys indicated that, while the domains, standards, and measures in the MHVQAS seemed relevant across models, LIAs sometimes had difficulty relating the requirements of the tool to the specifics of their model, particularly in regards to the types of documentation to provide. Reviewers said it would be helpful to have more guidance for scoring when a MHVQAS requirement is not part of a model. Additionally, participants commented that the MHVQAS included measures that were not part of their model, which was seen as a positive and a negative. As a positive, it could provide opportunities for improvement outside of the model standards. As a negative, it could feel like greater scrutiny and it could be very time consuming to meet the requirements in addition to the model requirements.

**Fig. 3:**
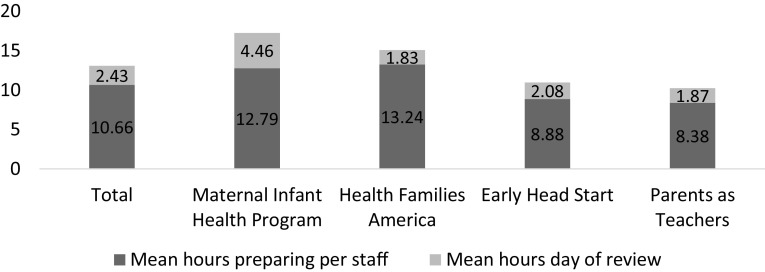
Mean hours staff spent preparing for and participating in the review process (n = 68)

Analysis of Variance showed that there was a not a statistically significant difference between the four models on time spent, the cost of time spent, or the total cost of preparing for and participating in the site review (see Table 4; Fig. 3).

**Table 4:** Differences in cost of the MHVQAS across home visiting models

	Mean	Min	Max	ANOVA		
				d.f	*F*-value	*p*-value
Hours spent per person (n = 68)	13.09	0	58	3	0.865	0.464
Cost of time per person (n = 62)	$346.57	$0.00	$2154.73	3	0.581	0.630
Total LIA cost (N = 8)	$2722	$1166	$4204	3	2.160	0.235

Altogether, reviewers spent an average of 28.90 total hours on preparing for and conducting site reviews, and preparing the Quality Report. Analysis of Variance showed that there was not a statistically significant difference between the four models on total time spent for reviewers, *F*(3, 8) = .512, *p* = 0.685.

## Discussion

The findings suggest that the MHVQAS can be applied across models, but would benefit from clarification of expectations for models that do not specify requirements in key areas of the tool. While the results showed quite a large degree of variation in performance across LIAs, no one model outperformed or underperformed others. Additionally, validity was not different by model, suggesting that the MHVQAS measures key components of quality in home visiting regardless of model. Ratings of satisfaction with the process by both reviewers and LIAs were also consistent across models, as were estimates of time and cost by both LIAs and reviewers.

Measures of reliability were different across some models. Interrater reliability was somewhat higher for HFA LIAs and somewhat lower for PAT LIAs. This finding may reflect differences in the extensiveness of these two models’ expectations for documentation and existing review procedures. While the study found no differences by model in LIA staff perceptions of reliability, reviewers felt that reliability was lower for MIHP as compared with HFA and PAT. As a Michigan-developed home visiting model, MIHP’s standards do not include expectations in some key areas of the MHVQAS tool, such as caseloads or dosage, and the MHVQAS tool does not set expectations for Michigan LIAs in these areas outside of what models require. Reviewers noted that this created ambiguity in the review process that could lead to inconsistent findings. This difference in perceived reliability can be addressed by offering clarification across measures regarding state expectations for models that do not specify requirements in key areas of the tool.

When compared with model developer reviews, the MHVQAS included a very comprehensive set of standards. The comprehensive nature of the MHVQAS was considered both a strength and limitation of the tool. It provided an opportunity to learn about strategies for assuring quality home visiting programs beyond the constraints of a model, and it highlighted new opportunities for improvement. However, it felt burdensome for some LIAs, and both reviewers and LIAs noted that it was challenging to know how to meet a standard when it is not part of a particular model. This challenge could be addressed by providing clearer guidance on state expectations when a specific model does not have expectations in a particular area. One gap in MHVQAS standards that was highlighted by the cross model standard comparison was Family Rights, which could be addressed by building in a standard related to this aspect of quality.

Both reviewers and LIA staff noted that one way to support all models in completing the MHVQAS efficiently would be to develop guidance that aligns model-specific documentation with specific measures on the MHVQAS. LIA sometimes found it challenging to recognize how the requirements of their model fit with MHVQAS standards and measures. Developing such a resource could improve the efficiency of the process.

Implementation quality drives outcomes in home visiting programs (Carroll et al. 2007; Donelan-McCall et al. 2009; Russell et al. 2007), and ensures effective and efficient use of programming dollars (Fixsen et al. 2005). States responsible for administering grant funding to home visiting programs are well-positioned to ensure that funds are directed toward agencies that implement quality programs and produce positive outcomes. States can also establish system-wide expectations for quality and fidelity, and support for quality improvement. However, few tools exist to support states in developing quality assurance and quality improvement tools and procedures that work across a home visiting system and the different models within that system. Research on building practical systems for this level of monitoring and improvement is also lacking.

The results of this study indicate that the MHVQAS shows promise as a tool and process to monitor implementation quality of home visiting services and inform decision making across models. These findings were used to inform revisions to the MHVQAS, which were put into practice with a pilot set of MIECHV funded LIAs in the spring of 2018. The pilot will be used to inform improvements to the tool, and to identify opportunities for individualized technical assistance, common professional development needs across LIAs, model implementation challenges, and best practices. While NFP did not participate in the study, most NFP programs in Michigan receive MIECHV funding, and will be required to participate in MHVQAS reviews. Based on the study finding that the tool performed comparably across four different models, we anticipate a similar result with NFP. Michigan is also considering using the MHVQAS across all evidence-based home visiting models, regardless of funding source, to facilitate development of statewide supports and technical assistance.

MIECHV funded states will benefit from tools and procedures that support their efforts to monitor implementation across evidence-based home visiting models. The MHVQAS offers a validated tool that will support states in meeting MIECHV’s expectation that states provide quality home visiting services to vulnerable families. The Michigan Home Visiting Initiative (MHVI) will be ready to share copies of the MHVQAS for use by other states beginning in fall 2018. Please contact the MHVI (MDHHS-HVInitiative@michigan.org) for a copy of the tool and to receive training on implementing the MHVQAS.

## Limitations

This study has several limitations. Using model reviews to measure criterion validity was challenged by the differences between each review’s specific measures and rating systems. The reviews did not have a one-to-one alignment at the measure level or between rating systems, which required more loosely looking at alignment at the standard level. Also, differences in ratings could have been attributable to a validity problem with the MHVQAS or to either improvement based on model review findings or drift from model expectations following the model review.

The study also had a very limited sample size that included LIAs selected for their experience implementing their model. A larger or more representative sample may have different perceptions of reliability and validity, different costs to participate, and potentially more variation between models. As such, it will be important to continue to collect data on key aspects of MHVQAS performance as it is implemented in practice to assure that the findings of this study hold true when a broader set of LIAs is assessed using the tool.

Finally, while an NFP representative participated in the design of the MHVQAS, the study cannot speak to how the MHVQAS performs in the context of an NFP program, because no NFP LIAs agreed to participate.
