# Correction to: Monitoring Quality Across Home Visiting Models: A Field Test of Michigan’s Home Visiting Quality Assurance System

**DOI:** 10.1007/s10995-018-2617-8

**Published:** 2018-08-22

**Authors:** Julia Heany, Jennifer Torres, Cynthia Zagar, Tiffany Kostelec

**Affiliations:** 1 Michigan Public Health Institute, Center for Healthy Communities, 2342 Woodlake Drive, Okemos, MI 48864 USA; 2 Michigan Public Health Institute, Public Health Services, 2364 Woodlake Drive, Suite 180, Okemos, MI 48864 USA; 3 Michigan Department of Health and Human Services, 109 W. Michigan Ave, 7th FL, Lansing, MI 48933 USA

## Correction to: Maternal and Child Health Journal 10.1007/s10995-018-2538-6

The article “Monitoring Quality Across Home Visiting Models: A Field Test of Michigan’s Home Visiting Quality Assurance System”, written by Julia Heany, Jennifer Torres, Cynthia Zagar and Tiffany Kostelec, was originally published electronically on the publisher’s internet portal (currently SpringerLink) on 05 June 2018 without open access. With the author(s)’ decision to opt for Open Choice the copyright of the article changed on 20 July 2018 to © The Author(s) 2018 and the article is forthwith distributed under the terms of the Creative Commons Attribution 4.0 International License (http://creativecommons.org/licenses/by/4.0/), which permits use, duplication, adaptation, distribution and reproduction in any medium or format, as long as you give appropriate credit to the original author(s) and the source, provide a link to the Creative Commons license and indicate if changes were made.

The original article has been corrected.

